# Identification of starting points to promote health and wellbeing at the community level – a qualitative study

**DOI:** 10.1186/s12889-019-6425-x

**Published:** 2019-01-16

**Authors:** Jennifer Hilger-Kolb, Claudia Ganter, Maren Albrecht, Catherin Bosle, Joachim E. Fischer, Laura Schilling, Claudia Schlüfter, Maria Steinisch, Kristina Hoffmann

**Affiliations:** Mannheim Institute of Public Health, Social and Preventive Medicine, Medical Faculty Mannheim, Heidelberg University, Ludolf-Krehl-Str. 7-11, D-68167 Mannheim, Germany

**Keywords:** Community health, Health promotion, Qualitative study, Community-based-participatory research

## Abstract

**Background:**

As health is influenced by the social, economic and environmental conditions in which individuals live, local communities are an ideal setting to promote health and wellbeing. However, up to now various health promotion interventions at the community level have had limited success, perhaps related to an incomplete understanding of local contexts and priorities. We therefore aimed to develop a broader and deeper understanding of topics or issues that were most salient to residents of a South-West German community by exploring their perceptions of needs, challenges, barriers and existing resources related to health and well-being.

**Methods:**

As an initial step of a multi-year community-based participatory research project, we conducted semi-structured interviews with key informants (*n* = 30) from various community settings (e.g., child care, elderly care, businesses, non-profit organizations, village councils, and local government). The terms “health” and “wellbeing” were included in the stem of each question in the semi-structured interview guide to enable a focus on related perceived needs, challenges, barriers and existing resources. Interviews were audiotaped, transcribed verbatim and analyzed using qualitative content analysis techniques.

**Results:**

Themes emerging from our interviews appeared to center primarily in three distinct areas: natural resources and built environment, access to services, and social cohesion including subthemes on the importance of social engagement and volunteerism, sense of community, and shared identity.

**Conclusions:**

That health and wellbeing were not identified explicitly as a priority by key informants suggests that these should not be presented as the primary focus of a community-wide initiative. Instead themes with a higher priority should be addressed in ways that can lead to better health and wellbeing as a secondary goal.

**Electronic supplementary material:**

The online version of this article (10.1186/s12889-019-6425-x) contains supplementary material, which is available to authorized users.

## Background

Health is determined by biomedical factors and the social, economic and environmental conditions in which individuals live [1, 2]. Public health researchers have long realized that the features of the communities in which people live often play an important role in promoting health and wellbeing [3].

Efforts to promote health within local communities have suggested the value of developing and implementing programs across sectors and within settings (e.g., schools, neighborhoods, work places, social networks and media) [4, 5]. Additionally, following the principles of the “community based participatory research” (CBPR) approach have shown promising results in promoting health within local communities [5–9]. CBPR is defined as a collaborative partnership between community members, organizational representatives, and research institutions that involves community members at various stages of the project such as setting priorities, decision-making, planning, and the implementation of health promotion strategies [6]. Previous research has shown that CBPR approaches can lead to more effective health promotion programs due to greater efficiency, sustainability and a more equitable distribution of services [2, 6, 8, 9].

One strategy often used in the early stages of CBPR is to identify barriers, needs, existing resources and opportunities related to community health through talking to key informants [10, 11]. A major strength of such a qualitative approach in CBPR is that responses are generated by participants and better reflect their own perspectives rather than, for example, the predetermined response categories found in a quantitative questionnaire. The result is often a deeper and broader understanding of health-related topics that are salient for community members [10, 12]. Previous work suggests that a better understanding of local contexts, a focus on identifying and overcoming barriers, and uncovering existing resources that could be strengthened to increase community health, are essential in enhancing the success of health promotion programs at the local level and in promoting their sustainability over time [5, 13].

Therefore, the objectives of our qualitative study were to obtain a broader and deeper perspective of topics or issues that were most salient to residents of a South-West German community by exploring their perceptions of needs, challenges, barriers, and existing resources within the community related to health and well-being. We anticipate that this comprehensive assessment approach will be useful in identifying appropriate community-endorsed starting points for promoting health and wellbeing in both the target community and other settings.

## Methods

Our study represents an early component of the One Good Year Added (OYA) study, a multi-year CBPR project led by the Mannheim Institute of Public Health. Our target community is located at the Western edge of the Black Forest, Germany. The target community consists of a center city with eight affiliated villages or suburban areas administered by a single mayor and local government. In addition, each affiliated village/suburb has its own village council elected by the residents of each village/suburb to represent their interests in the local government of the target community. In total, the target community has approximately 30,000 residents. The community is bisected by a river along which several industrial companies are located including a car manufacturer that employs around 6.500 workers. As the community represents a typical mid-sized South-West German community and the mayor has intended to establish health promotion strategies within the city, the community seemed to be the ideal place for starting the OYA project. The overall aim of the OYA project is to promote health and wellbeing in ways that would result in the addition of (a) healthier year(s) of life for all community members. The project received ethical approval by the Medical Ethics Committee of the Medical Faculty Mannheim, Heidelberg University (2016-588 N-MA).

### Definition of the term community

The meaning of the term “community” varies across research disciplines [14]. We understand “community” in both geographical and social terms in which residents of a politically organized entity share concerns and responsibilities for issues of common importance such as health promotion and disease prevention [14].

### Definition of the term setting

In line with the definition used by the World Health Organization (WHO), we considered a “setting” a *“place […] in which people engage in daily activities in which environmental, organizational and personal factors interact to affect health and wellbeing”* [15].

### Participants

In preparation for the OYA project, the following structures were established to plan and implement health promotion interventions in the community: a steering committee, a scientific sounding board, and a community sounding board. The steering committee consists of the principal investigator (JEF), the mayor of the community, the coordination office (project administration from the community site and project administration from the Mannheim Institute of Public Health [CS, MA]). The scientific sounding board is represented by researchers from various disciplines that provide the steering committee with evidence-based information on different topics related to community health. The community sounding board is comprised of a rotating membership of residents that represent different community “settings” (e.g., child care, elder care, businesses, non-profit organizations, village councils, and local government). These community sounding board members represent the interests of the residents because they either belong to a special population group (e.g., elderly), were representatives of facilities or organizations (e.g., churches, schools, sports clubs), or were elected by the community members to a post (e.g., village councils). As community sounding board members represent the views of the community members from various settings, they served as the key informants for the current qualitative assessment. In line with the CBPR approach, key informants (e.g., from community sounding boards) can assist the researchers in identifying priorities and enable better access to community residents. According to McKenna, et al. [11] another advantage of using members of a community sounding board as key informants is that they will be directly involved in the planning of health promotion interventions later on in the project. Besides being a member of the community sounding board, no further inclusion and exclusion criteria were specified to take part in an interview.

Recruitment for the qualitative interviews began in August 2016 using a mail invitation sent by the steering committee in which general information about the project was provided. Follow up contact with each individual on the community sounding board was made by phone call or email one week after the initial invitation. Those who could not be reached after three attempts were considered non-responders. We contacted a total of 37 community sounding board members with 30 agreeing to participate (response rate: 81.1%). As concurrent theme analysis (described below) suggested theme saturation following interviews with this sample, we chose not to expand recruitment to individuals outside the community sounding board. As further confirmation of theme saturation, however, as mentioned before, we invited all members of the community to a participatory workshop to hear their views on the topic “Increasing health and wellbeing within the community”.

### Semi-structured interview guide

Potentially relevant topics related to health and wellbeing in the target community were identified during a two-day workshop that took place at the target community before the official start of the OYA project. The workshop was attended by the steering committee, the scientific sounding board and various members of our research institute. The aim of the workshop was to get to know the target community and its main characteristics. The workshop included district inspections by foot, visits to various settings (e.g., kindergartens, schools, nursing homes for elderly, and companies) and summary reports about the observations made in the community. Based on the results of the workshop three of the authors (CG, JEF, KH) developed the semi-structured interview guide with open-ended questions to explore the following three main topics in the interviews: resources that were already present in the community and facilitated the promotion of health and wellbeing; existing barriers that currently worked against health and wellbeing; and needs and challenges that should be addressed to promote health and wellbeing in the community (Additional file 1). To assure interview questions did not lead the interviewee in a pre-defined direction and to gain comprehensive information, we developed both broad and detailed interview questions. To assure that we do not miss any relevant theme, a final question probed other issues/topics potentially relevant to promoting health and well-being in the community that had not been raised during the interview. Feedback from all authors was provided and incorporated into the interview guide. In line with the CBPR approach the interview guide was also discussed with and approved for use by the steering committee at the community site.

### Interview procedure

As an initial step, two interviews were conducted by two of the authors (CG, CS) to pretest the applicability of the interview guide. As only minor changes in the word order of two interview questions were made and no differences in interview style were observed, we included data from both test interviews in our analysis.

Separate face-to-face interviews with each interviewee were conducted in German from August to November 2016 by three of the authors (CG, CS, MA). All interviews were conducted in private settings in the absence of others. The majority (*n* = 27; 90%) took place in person and on-site in the community with three interviews conducted by phone at the interviewees’ request. At the beginning of each interview, interviewees were encouraged to respond from the perspective of the population group or setting that they represented. To reduce social desirability bias, interviewees were assured prior to the interview that there were no right or wrong answers. Interviewees were advised that their participation was voluntary, written informed consent was obtained in each case and no compensation was provided.

### Data management and analysis

All interviews were audiotaped (Olympus-DS-2500) and transcribed *verbatim* by an external transcription service. Each transcription was checked for accuracy and anonymized by the research team. Transcripts averaged 22 pages (min. 10; max: 38) in length.

Transcripts were loaded into MAXQDA 12.3 (VERBI Software GmbH, Berlin), a qualitative data analysis software package that assisted in interview coding and analysis. Structuring qualitative content analysis suggested by Kuckartz [16] was used to analyse the data. In a first step, all authors developed an initial coding scheme based on the topics addressed in the semi-structured interview guide including the following main categories: “Existing resources”, “Existing barriers”, “Needs and challenges”, and “Further topics”. The applicability of the initial coding scheme was tested by all team members coding two interviews independently. In a second step, the resulting coding scheme was applied to all interviews and was continually refined through identification of inductive (sub)codes that emerged out of the interview material during the coding process. Any new codes were discussed and adopted during regular team meetings only if all coders agreed. In a third step all interviews were coded again by applying the final coding scheme. In a final step all coded interviews were assessed for emerging themes and subthemes presented in the result section.

For quality assurance in data coding, we used several strategies. Interview transcripts were assigned to five coders (CB, JH-K, LS, MA, MS) who worked in pairs. Each pair independently coded a set of two to three interviews and then compared coding assignments. Differences in coding assignments were discussed until consensus was achieved. The composition of coding pairs was changed after two to three interviews to reduce the possibility of introducing a systematic bias.

The notes that were derived from the participatory workshop with community residents were transferred into MAXQDA and analysed applying the same methods used for analysing the qualitative interviews.

## Results

In total, 30 individuals participated as key informants. They had a mean age of 53.0 years (range: 29–72 years) and the majority were male (*n* = 20). In responding to prompts to identify resources for health and wellbeing, a theme commonly addressed by various interviewees centered on the presence and importance of *natural resources* and *features of the built environment*. Interviewees consistently mentioned green spaces surrounding the community as a vast resource for enhancing quality of life and remaining physically active.

*“[…] if you say ‘I want to go walking in the woods’ then you can be there within 10 minutes, no matter where you are in [Community name]. For sure, that is quality of life.” (ID 26; Local government representative)*

There was general agreement that these resources (e.g., walking paths, public swimming pools) should be renovated, maintained or enhanced, providing further evidence of their value to members of the community.

*“[…] and if I say: ‘Okay, if I wanted to have a healthy city’ then I would expect walking and hiking paths that are well connected to all community districts, are well maintained, and appropriately signposted.” (ID 3; Local government representative)*

Moreover, most interviewees seemed *proud of many existing resources*, felt that these were somewhat unique in the region and made life in the community more enjoyable and richer.

For example, interviewees commonly emphasized satisfaction with services in the community such as those providing care for children, adolescents and senior citizens.

*“I think the nursing homes for elderly and other elderly care services here in [Community name] -and I know a lot of cities- are already exemplary. I think that caregivers [in these facilities] do a lot to keep the elderly physically and also mentally active” (ID 15; Church representative)*

Interviewees also commonly felt that the high quality of these services was dependent on *social engagement and volunteerism* within the community. Specifically, the high level of support for various community subgroups like children, the elderly and refugees and the existence of a broad spectrum of associations such as sport clubs, choral societies and gardening societies in every city district was endorsed as essential by many interviewees and in some cases suggested as a mechanism for promoting social equitability.

*“Well, I think that [Community name] without social engagement things would not work. […] It’s really amazing! [Community name] can be proud of all the volunteers.” (ID 26; Local government representative)*

*“What makes us [community] special, of course, is the diversity of clubs. […] This is, I think, very, very important, not only as societal and social aspects, but also with regard to maintaining health and supporting the interests of each citizen. People are very, very engaged.” (ID 8; Local government representative)*

As with expressed interest in maintaining or enhancing features of the built environment, interviewees endorsed the need to further *enhance the quantity or quality of some services* in the community (e.g., extending hours of operation and offering weekend services at day care facilities to better fit parents work schedules) as an opportunity for *building on existing resources*. For example, interviewees identified that having a higher caregiver/child ratio might better assist children with special needs, provide a more stimulating learning environment for children with particular interests, and reduce teacher burden in the classroom.

*“Expanding care time [for children]. Also more diverse [hours of operation]/perhaps the different facilities could provide different offers to enable [the parents] to say ‘Okay, there is also better afternoon care or also additional care at the weekends’.” (ID 36; Employer)*

*“Additionally, the workload for the caregivers would be much lower [due to fewer children attending one class]. It would also allow structuring the pedagogical work in a completely different way.” (ID 8; Local government representative)*

Interviewees specifically identified a need to *enhance services for the elderly* in surrounding suburbs by providing opportunities for assisted living or home-based nursing care. Some felt these services were essential for increasing quality of life in this age group and for offering senior citizens a choice for remaining in a familiar living environment.

*“Well, I think for senior citizens it would be of high priority to stay in their familiar environment as long as possible. […] In particular, we are strongly attached to our community districts, our villages. But so far, only a few age-based living opportunities or age-based care services exist in these districts […]. And I think that is something that is very important for the elderly.” (ID 1; Local government representative)*

Moreover, interviewees identified the *centralization* of other services (e.g., medical care, pharmacies, shopping) in the city center as a challenge – not only for the elderly but also for other residents living in outlying communities without regular public transport. Some interviewees stressed the need for introducing shuttle busses or car pooling to ensure equal access to these services for all community members.

*“There is no physician, there is no pharmacy, not only here [name of the suburb], but also in other suburbs [except for the city center]. And of course, that is a deficit.” (ID 20; Village council representative)*

*“That [barriers to health and wellbeing] are things like you can’t buy groceries in every suburb. There are suburbs in our community where you have to rely on public transport which is also not very good everywhere. That is due to the fact that almost everyone has a car and nobody goes by bus […]. But it has a negative effect for those who are not mobile. And then, of course, when you're not so mobile anymore, it becomes a problem, when there is not even a bakery in the suburb. And of course, we also have such suburbs where you have to drive [to the center] to do your grocery shopping.” (ID 1; Local government representative)*

*“For example, some people are not so mobile anymore. They say: ´Well, I still drive to the city center, but not in the evening anymore, and there is no transportation option´, and we respond: ´Do you know what? We'll pick you up, we'll bring you home.´ Offering such things is very important, because these people, for example, they would otherwise sit at home alone […].(ID 22; Local government representative)”*

In addition to better service accessibility, the need for more *effective outreach of existing services* to specific age groups in the community was stressed by some interviewees. For example, offering more activities targeting adolescents and the elderly were identified as valuable for improving quality of life in these age groups by some interviewees, however views on this topic differed. Some felt, for example, that adequate opportunities already existed while others emphasized that new approaches should be developed to better reach adolescents and elderly people.

*“In most suburbs, there is a trailer or something else where the youth have the chance to get together. And in the city center […] the central meeting point is the Youth- and Family Center.” (ID 13; Child care representative)*

*“There are no afternoon programs for adolescents aged 12 to 18. That is another shortcoming of the whole community. […] They [the adolescents] are left alone, they hang around on school yards or anywhere else – and wherever they are others feel bothered by them.” (ID 20; Village council representative)*

*“With regard to the elderly, I’m aware of church associations that have introduced the so-called pensioners’ clubs about 30 years ago, where the elderly can meet one afternoon per week. That’s an opportunity for the elderly to get together occasionally and without obligation. That is very well accepted.” (ID 26; Local government representative)*

*“One gets the impression that those pensioners’ clubs have become outdated. Well, the format of meeting in the afternoon, it no longer reaches the target audience.” (ID 14; Church representative)*

Offering places where *social contact* across generations and between those from differing cultural backgrounds was voiced as a related need for increasing quality of life in the community. Some interviewees expressed this as a desire for greater *community solidarity* – that resources were developed in ways that allowed them to reach all residents throughout the community. One suggestion, for example, was that leisure time activities or events be offered at low or no cost to avoid excluding those with limited resources. Similarly, ensuring barrier-free access for the disabled, the elderly and families with small children was emphasized. Although interviewees acknowledged current efforts in this direction, some noted that additional infrastructural changes (e.g., barrier-free access to public buildings, public swimming pools, and other cultural activities) remained nonetheless necessary to allow more equitable access to all community members.

*“Offering activities for free to the population, that would allow for example, single, elderly persons on a limited budget to participate. I think that is also very important.” (ID 22; Local government representative)*

*“Still, a lot has to be done in the field of inclusion. And there are certainly many things that were not taken into consideration but can be implemented without any financial investment, and I think with regard to those things we are doing really well. But if we talk about creating barrier-free access in the whole infrastructure, a lot of funding is required.” (ID 1; Local government representative)*

Some interviewees emphasized that *a sense of community, intact social structures, common experiences, and social cohesion* were essential elements for living longer, being happy and staying healthy.

*“The question is: Does our attitude to life, our sense of community, our social life work out or not? For me, the factor of feeling good – the common experiences and the feeling of being supported by a social network – for me that is the biggest factor for living a long, happy, and healthy life.” (ID 30; Local government representative)*

However, concerns regarding a declining trend in *solidarity and social cohesion* within the community was recognized as a challenge particularly in the areas of social engagement and volunteerism. Young adults, for example, were viewed as less motivated to join or participate in sports clubs or other local associations. In reflecting on this challenge, some interviewees observed that expectations and attitudes around participation in social events may have changed over time, perhaps due to increased job demands, a growing sense of job insecurity and increased mobility. Some also observed that community organizations had been confronted with the spiraling consequences of decreased social engagement for the first time through decreasing membership and fewer resources to maintain existing activities. Other interviewees felt that more support from the local government would be helpful in motivating volunteers to ensure social engagement in the future or at least preserve the status quo.

*“[…] the young generation, that should grow up taking over the volunteer work in a club or something - it is no longer like it was 30 or 40 years ago. This is due to leisure time activities and also mobility, [...]. Today, young people have a car they are mobile, they can do whatever they want, and thus get less involved in local clubs or such things [...]” (ID 31; Local government representative)*

*“[…] that is something that will affect us here in [cityname] even more in the future, because we notice that the clubs are suffering. A lot of clubs, especially the choral societies they will no longer exist in the near future because they do not attract new members.” (ID 24; Club representative)*

*“There are no trainers or let’s say not enough trainers who are also available during the day [only in the evening]. And that is something really difficult for some clubs” (ID 23; Club representative)*

*“What I feel is very important for example, that when social engagement takes place, things like [Name of events in the community] or anything else, then it has to be the task of the city to provide an infrastructure for this [...]. And I think that's what the city [Community name] has to do, because if it wants that its citizens do something, then it [the city] has to support its citizens” (ID 22 Local government representative).*

*“But maybe, as we are already talking about it, one of the possible levers that should be considered, how can the city even more than today, also more structurally, help the clubs, I would say, help the clubs help themselves, in principle. Maybe just starting any activities or offering support from the city, that makes it easier to keep the current club structure alive. For me, that is actually one of the essential: So that what [the activities] we have today, no additional ones, but what we have today, at least preserve the status quo. And there are, of course in the near future, schools that will probably not be able to replace the club activities, and even less in the area of elderly care.” (ID 29 Village council representative)*

Although some interviewees expressed an interest in initiatives that fostered a greater *sense of community* and *shared identity*, they recognized the issue was a complex one. Some interviewees stressed that the strong social identification individuals had to their suburbs was a challenge for establishing a common identity throughout the larger community. At the same time, some also felt it important to respect and possibly leverage the diversity of social structures and sense of shared identity that existed within the suburbs as a useful strategy for initiating a project to bring all members of the community together toward a common goal.

*“[…] there is the city and there are villages around it. But this, together an identity of [Community name]. If you would ask someone here at [name of the suburb]: `Are you [name of the community]?`; He would probably answer: `No, I’m [name of the suburb]´. And to break this up in a positive sense, like Hebbel, he merges opposites to something new but preserves the peculiarities […]. And I think that’s what many people want. And the potential is there.” (ID 14 Church representative).*

*“This brings us back to the identity of the whole community. [...] Promoting a shared identity and bringing it forward would be an important aim, but without destroying the intact local structures in the suburbs. [...] That reminds me that this is almost the same as on the European level. How can we bring European politics forward while at the same time preserving individual identities? […] It is almost the same in the small universe of our community.” (ID 30; Local government representative)*

In summary, themes that emerged during our interviews centered mainly around three distinct areas: natural resources and build environment, access to services, and social cohesion (Table 1). The themes and subthemes identified from our analyses of responses from the participatory workshop involving members of the general public suggested a high degree of overlap with the themes emerging from data provided by key informants. Specifically, we found little evidence of additional barriers, resources, opportunities or priorities in the target community.

**Table 1:** Summary of themes/subthemes identified in the qualitative interviews

*Natural resources and built environment*	
• Enhance these resources	
• Proud of existing resources and their quality	
• Build on existing resources	
*Access to services*	
• Enhance services for specific subgroups	
• Centralization of services	
• Improve public transport	
• Effective outreach of existing services to specific age groups (e.g., adolescents, elderly)	
*Social cohesion*	
• Offering places were social contact is possible	
• Community solidarity	
• Social engagement and voluntarism	
• Shared identity	

## Discussion

### Main findings

We conducted a comprehensive assessment among key informants to uncover needs, challenges, barriers and existing resources that might help identify community endorsed starting points for efforts to promote health and wellbeing in the community and should be considered in the planning and implementation of such initiatives. The themes that emerged were not directly related to either health or community wellbeing and appeared to center on what one might consider more “upstream” determinants of health and wellbeing [17]: *natural resources and built environment, access to services,* and *social cohesion* including the subthemes *social engagement and volunteerism, sense of community,* and *shared identity*. That health and wellbeing were not identified as specific priorities by key informants suggests that these should not be presented as the primary focus of a community-wide initiative, but rather be incorporated as secondary goals in future programming.

### Possible starting points

Given that interviewees consistently endorsed the importance of *natural resources*, it might be useful *building on these resources* in future efforts to increase health and wellbeing. For example, modernizing existing walking paths in local forests or building new ones were suggested as one possibility to foster physical activity among community members – a common health promotion goal. Furthermore, better leveraging local forests as recreational resources may also improve physical and mental health, as previous research has shown associations of the natural environment (e.g., parks and green spaces) on both health outcomes [18–20].

*Building on specific, well-functioning resources* identified by interviewees (e.g., services for children and the elderly) also appears to be a strategy that would work in the target community. Enhancing the quality of care provided in institutions like day care facilities, schools and also nursing homes (e.g., extending hours of operation or increasing the caregiver/child- or the nursing staff/resident-ratio) was endorsed as an effort that might translate into better wellbeing and quality of life in children and the elderly and has also been found in former research [21–23]. In addition, such improvements were thought to reduce the burden on caregivers, nursing staff, parents, and family members caring for dependent relatives resulting, in turn, in greater job satisfaction in caregivers [24–26] and a better quality of life in parents and family members [27–29].

Future programs could also benefit from *volunteerism and social engagement*, as these were consistently mentioned as central resources in the community. A useful strategy prior to implementing programs to increase health and wellbeing might be to leverage these resources by involving already engaged community members or to build partnerships with existing social or sports organizations [30]. This approach has the benefit of capitalizing on the knowledge and expertise in what works well in planning and implementing any new undertaking in their community. Involvement by community residents may also prove valuable in reducing reluctance among other community members in participating in newly developed programs (e.g., the “snowball effect”) [31].

However, as interviewees identified the challenge of sustaining social engagement over time, developing strategies to accomplish this goal and increase the attractiveness of volunteerism in the community seem necessary. Local employers, for example, could contribute to the sustainability of social engagement and volunteerism by encouraging employees to engage socially outside their paid work [32]. For example, offering flexible working hours might facilitate social engagement among employees. Puska [30] also suggested including various organizations from different sectors and settings into the project as a useful strategy to successfully implement health promotion programs at the community level. In addition, he emphasized that it was important to establish such collaborations as “win-win situations” for both sides, the community and the organizations [30]. In addition, key informants stated that receiving more support from the local government was important for motivating volunteers and for ensuring the existence of clubs in the future. However, it seemed that besides a suggestion for waiving utility costs, community members shared few concrete ideas about the shape such support strategies might take.

Interviewees commonly identified the centralization of and access to services as a fundamental barrier in the community. Penchansky and Thomas [33] identify five dimensions of access that should be carefully considered in program planning: *availability, accessibility, accommodation, affordability, and acceptability*. While the *availability* of services (e.g., the volume and type of existing services such as medical care, grocery shopping, or cultural activities) was commonly endorsed as a valuable resource in the city center, improving the *accessibility* of these services for residents living in the suburbs was identified as a need. An initiative that included on demand shuttle services to the city center might represent an appropriate initial response, for example. Indeed, some interviewees suggested that such a shuttle service could improve quality of life among various community members that have to rely on public transport to get to the city center (e.g., children, adolescents, and the elderly). Extending transportation networks to ensure greater accessibility of locations where leisure-time activities take place were thought to address two emergent themes: an opportunity for equal use of existing resources like woodland parks to a larger segment of the community who might otherwise lack direct access to a natural resource and a boon for enhancing quality of life. In a former CBPR study missing transportation networks were also suggested interviewees as a barrier to participate in physical activity or to buy healthy food [11]. Developing informal transportation networks in the form of community car pooling and modifying the built environment to ensure accessibility for the elderly or disabled represents additional strategies endorsed by interviewees as potential starting points.

Offering services occasionally in the suburbs outside the city center represents a third strategy identified by interviewees that might address both limited accessibility and *accommodation* - the extent to which services are organized to accept and act on input from users, including the perceived appropriateness of aspects of these services (e.g., hours of operation) [33]. Implementing programs that incorporate this strategy might be quite effective in promoting health. A systematic review documents, for example, that offering mobile markets, conducting weekly farmer’s markets, or installing fruit and vegetable stands in the suburbs increases access to and consumption of fresher, more nutritious foods [34, 35]. In line with previous research key informants emphasized that *affordability* should also be considered when planning future programs [11]. For example, offering such programs at no or low costs, were suggested as a way to enable community members on a limited budget to participate in future programs. Although views on a fifth dimension of access, the *acceptability* of services (e.g., user attitudes on provider characteristics and provider attitudes on user characteristics) [33] were varied, creating greater acceptability of services to better reach specific, often underserved age groups such as adolescents and senior citizens in this community might represent a future focus.

Beyond questions of access to services within their community, many interviewees voiced concerns regarding decreased social contact and the relative absence of gathering places for fostering cross-generational and cross-cultural social exchange. These concerns might be addressed in health promotion initiatives. Previous work suggests, for example, that the activity of planning and establishing such a gathering place could, by itself, serve to enhance a sense of community and foster a shared identity, and may also increase health and wellbeing of community members [36]. For the success of such a project, outreach and active engagement by members throughout the community and their involvement in the planning and implementation processes appears essential [30].

### Limitations

Although this study uncovered several emerging themes salient to members of the target community with potentially broader implications elsewhere, a few limitations should be acknowledged. First, we restricted interviews to members of the community sounding board. While these individuals came from or officially represented a variety of different community settings, their views on resources, existing opportunities or barriers might have been unique or failed to reflect the potentially broader views of community members. To ensure a breadth of views, we obtained and analyzed data from members of the general public attending our participatory workshop on increasing health and well-being in the community. Second, as we did not conduct a formal process of member checking, our interpretations may have differed in important ways from the perspective of our interviewees. We observed, however, a high degree of consistency in the themes and subthemes emerging from key informant interviews, suggesting theme saturation and further confirmation of this by data from community attendees at the participatory workshop. We therefore consider our data to be useful in identifying potential starting points and key issues for consideration in planning and implementing a community-based participatory research initiative. Third, we focused on resources, barriers, challenges, and needs in a single community. Although the relative importance of these emerging themes might vary or the presence of other more pressing issues may exist in other settings, we feel that the overarching value of the strategy we used is that it can be widely applied in diverse settings to identify starting points that are salient to community members and that can shape efforts to promote health or other objectives in more locally meaningful and therefore effective and sustainable ways. Importantly, this approach benefits from the CBPR tradition in which insights and priorities are derived from members of the target community that might have been otherwise overlooked.

## Conclusion

Our study uncovered needs, challenges, barriers and existing resources that might identify starting points in the planning of a community-wide initiative to promote health and wellbeing. That health and wellbeing were not identified explicitly as a shared goal by key informants suggests that these should not necessarily be presented as the primary focus of a community-wide initiative. Rather, our analysis suggests the potential value of addressing themes with a higher priority in ways that can lead to better health and wellbeing as a secondary goal.

## Additional file

Additional file 1: Translated version of the interview guide. (DOCX 17 kb)

## Abbreviations

CBPR: Community based participatory research
OYA: One Good Year Added Study
WHO: World Health Organization
