# Supplementary material for: Identification of starting points to promote health and wellbeing at the community level – a qualitative study
Source: BMC Public Health. 2019 Jan 16;19:75. doi: 10.1186/s12889-019-6425-x (PMC6335792; doi:10.1186/s12889-019-6425-x)
Supplement: Supplementary file 1 — Translated version of the interview guide. (DOCX 17 kb) [file 12889_2019_6425_MOESM1_ESM.docx]

**Additional File 1: Translated version of the semi-structured interview guide developed for the key informant interviews of the OYA study**

- Can you please tell me a little bit more on your function in [name of the target community]?
  - How long have you been performing this function?
  - What are your main tasks?
  - Which subgroup within the community do you represent?
- If you think of [name of the target community], how would you generally describe the target community?
- The overall aim of the “One good year added” project is to promote health and wellbeing in ways that would result in the addition of (a) healthier year(s) of life for all community members. Please tell me about resources that already exist within the community to promote health and wellbeing of community members.
  - Why do you think these resources promote health and wellbeing?
  - Are there any specific resources for the subgroup that you represent? Which ones?
- Please think about programs that have been conducted within the community to promote health and wellbeing before the official start of the “One good year added” project? Can you tell me something about these programs?
  - What were the specific aim of these programs?
  - Which community members were addressed by these programs?
  - Which of the programs you named, has/have been successful? What do you think are the reasons for the success?
  - Which of the programs you named, has/have not been successful? What do you think are the reasons that the program/s has/have not been successful?
- Please think about existing barriers within the community that work against health and wellbeing? Please tell me something about these barriers?
  - Are there any specific barriers for the subgroup that you represent? Which ones?
  - Why do you think these barriers work against health and wellbeing?
  - What can be done to overcome these barriers?
- What are the needs to successfully promote health and wellbeing of all community members?
  - If you again think of the subgroup you are representing, do they have any specific needs? Please tell me more about these needs.
- Are there any challenges that may affect the aims of the “One good year added” project?
  - Which kind of challenges do you see?
  - Why do you think these things might be challenging?
- Are there any other issues or topics that might be relevant for the “One good year added” project that you want to tell me about?
- Are there any additional persons within the community we should talk to?
- May I contact you again, if I should have any further questions?
